# Chronic Glucocorticoid Exposure-Induced Epididymal Adiposity Is Associated with Mitochondrial Dysfunction in White Adipose Tissue of Male C57BL/6J Mice

**DOI:** 10.1371/journal.pone.0112628

**Published:** 2014-11-12

**Authors:** Jie Yu, Bing Yu, Jun He, Ping Zheng, Xiangbing Mao, Guoquan Han, Daiwen Chen

**Affiliations:** 1 Animal Nutrition Institute, Sichuan Agricultural University, Chengdu, 611130, People's Republic of China; 2 Key Laboratory for Animal Disease-Resistance Nutrition of China Ministry of Education, Sichuan Agricultural University, Chengdu, 611130, People's Republic of China; 3 College of Food Science, Sichuan Agricultural University, Ya'an, 625014, People's Republic of China; Faculty of Biology, Spain

## Abstract

Prolonged and excessive glucocorticoids (GC) exposure resulted from Cushing's syndrome or GC therapy develops central obesity. Moreover, mitochondria are crucial in adipose energy homeostasis. Thus, we tested the hypothesis that mitochondrial dysfunction may contribute to chronic GC exposure-induced epididymal adiposity in the present study. A total of thirty-six 5-week-old male C57BL/6J mice (∼20 g) were administrated with 100 µg/ml corticosterone (CORT) or vehicle through drinking water for 4 weeks. Chronic CORT exposure mildly decreased body weight without altering food and water intake in mice. The epididymal fat accumulation was increased, but adipocyte size was decreased by CORT. CORT also increased plasma CORT, insulin, leptin, and fibroblast growth factor 21 concentrations as measured by RIA or ELISA. Interestingly, CORT increased plasma levels of triacylglycerols and nonesterified fatty acids, and up-regulated the expression of both lipolytic and lipogenic genes as determined by real-time RT-PCR. Furthermore, CORT impaired mitochondrial biogenesis and oxidative function in epididymal WAT. The reactive oxygen species production was increased and the activities of anti-oxidative enzymes were reduced by CORT treatment as well. Taken together, these findings reveal that chronic CORT administration-induced epididymal adiposity is, at least in part, associated with mitochondrial dysfunction in mouse epididymal white adipose tissue.

## Introduction

Glucocorticoids (GC) are adrenal cortex-secreted steroid hormones stimulated upon hypothalamic-pituitary-adrenal (HPA) axis activation due to endocrine disorder and/or stress [1]–[3]. Patients with chronic exposure to excess GC, either resulted from Cushing's syndrome or receiving GC therapy for the purposes of anti-inflammatory and/or immunosuppression, develop numerous metabolic changes including obesity, fatty liver, dyslipidemia, and glucose intolerance [4]–[6]. Obesity is part of metabolic syndrome and shares with a number of other diseases common pathogenic origins like inflammation [7]. Hypercortisolemia-induced obesity is characterized by central adiposity caused by distributing fat accumulation from subcutaneous adipose tissue to the metabolically more active visceral fat [8], [9]. In addition, mice selectively expressing the glucocorticoid-activating enzyme, 11 β-hydroxysteroid dehydrogenase type 1 (11βHSD1), in adipose tissue shows central obesity phenotype [10], whereas the suppression of 11βHSD1 attenuates adipogenesis [11]. Though the lipogenic effect of GC has been well defined in the past decades, GC are also widely known to stimulate lipolysis through activating hormone sensitive lipase (HSL) and mobilizing nonesterified fatty acids (NEFA) to β-oxidation as “stress” hormones [12]–[14]. Most recently, it has been documented that GC simultaneously increase both lipolysis and adipogenesis through separate pathways [15]. The dual roles of GC in lipid metabolism, apparently, make the process through which GC exposure induces adiposity more elusive.

Mitochondria are double-membrane organelles that produce the majority of cellular ATP and reactive oxygen species (ROS) mediated by the mitochondrial-specific proteins [16], [17]. White adipocyte mitochondria support the anaplerotic generation of metabolic intermediates for fatty acid synthesis[18]. Decreased mitochondrial density and oxidative phosphorylation activity in white adipocytes have been shown in both rodent and human obesity [19]–[21]. Likewise, reduced mitochondrial DNA (mtDNA) copy number directly correlates with increased lipogenesis in white adipose tissue (WAT) [22]. Furthermore, enhanced mitochondrial biogenesis increases the fatty acids oxidation and decreases the susceptibility of high-fat diet-induced obesity [23], [24]. Thus, mitochondria play prominent role in WAT lipid homeostasis.

The objective of the present study was to test the hypothesis that impaired mitochondrial functions may contribute to chronic GC exposure-induced epididymal adiposity.

## Materials and Methods

### Ethics statement

All animal-related procedures were approved by Sichuan Agricultural University Institutional Animal Care and Use Committee (M2012-07-08). All surgery was performed under isoflurane anesthesia, and all efforts were made to minimize suffering.

### Animals, housing, and treatment

Thirty-six 5-week-old male C57BL/6J mice (∼20 g) that are originally purchased from Jackson Laboratory (Bar Harbor, ME, USA) were obtained from Sichuan Academy of Medical Sciences (Chengdu, Sichuan, China). Mice were group-housed (3 mice/cage) in standard polycarbonate cages at 21±2°C on timed 12-h dark, 12-h light cycles and had free access to standard rodent chow and tap water. After 1-week acclimation, animals were assigned into 2 treatments by body weight. According to previous report [25], 100 µg/ml of CORT (Sigma-Aldrich, St. Louis, MO, USA) was dissolved in ethanol, and then diluted in tap water to a final ethanol concentration of 1% (v/v). Mice were provided with drinking water containing 100 µg/ml CORT or 1% (v/v) ethanol solution alone (vehicle). During the experimental period, *ad libitum* diet remained available.

Animals were weighed weekly. After 4-week of the treatment, mice were anaesthetized and sacrificed. Blood was collected and centrifuged at 3000 g for 15 min at 4°C. Plasma samples were stored at −20°C for future measurements. Epididymal, retroperitoneal and inguinal subcutaneous WAT depots were dissected and weighed as previously described [26]. The tissue samples were frozen and stored at −80°C for further analysis.

### Histological analysis

The collected epididymal WAT was fixed in formalin solution, dehydrated, paraffin-embedded, and sectioned. Sections (10 µm thick) were stained with hematoxylin and eosin, then examined and photographed under Olympus Microscope Digital Camera DP21 (Olympus China, LTD., Beijing, China). Adipocyte sizes were measured for at least 200 individual cells per mice with Image J software (National Institutes of Health, Bethesda, MD, USA).

### Hormone and biochemical measurements

Plasma CORT concentrations were measured using a commercial radioimmunoassay (RIA) kit from MP Biomedicals Inc. (Solon, OH, USA). Plasma insulin, leptin contents were determined with specific mouse enzyme-linked immunosorbent assay (ELISA) kits (Millipore, Billerica, MA, USA). For plasma fibroblast growth factor 21 (FGF21), ELISA kit (BioVendor, Candler, NC, USA) was applied to measure the concentrations. All measurements were performed according to respective manufacturer's instructions.

The concentrations of triglyceride, NEFA and malondialdehyde (MDA) were measured using commercial assays (Jiancheng Institute of Bioengineering, Nanjing, Jiangsu, China) as described previously [27]. The activities of superoxide dismutase (EC Number: 1.15.1.1), gluthathione peroxidase (EC Number: 1.11.1.9) and catalase (EC Number: 1.11.1.6) were measured using specific commercial kits (Nanjing Jiancheng Institute of Bioengineering, Nanjing, China) according to the provided instructions, respectively.

### DNA and RNA extraction

Total RNA from frozen adipose tissues was extracted using TRI reagent (Takara, Dalian, China) and further purified with Qiagen RNeasy Mini kit (Qiagen, Valencia, CA, USA) [27]. Total DNA was isolated from epididymal WAT using a DNAiso Reagent (Takara). Concentration and integrity of the extracted RNA were determined with an Agilent 2100 Bioanalyzer (Santa Clara, CA, USA).

### Quantitative real-time PCR

Quantitative Real-time PCR was conducted as previous report [28], [29]. Briefly, the cDNA was synthesized from 1 µg total RNA using random primers and reverse transcriptase (Takara). Real-time quantitative PCRs were performed using Power SYBR Green PCR master mix (Applied Biosystems, Foster City, CA, USA) on an Applied Biosystems 7900HT real-time PCR system. The conditions for these PCRs were 40 cycles of 95°C for 15 sec and 60°C for 1 min. The real-time PCR of each sample were performed in duplicate. The data were analyzed using 18S rRNA as the internal control with the cycle threshold (2^−ΔΔCT^) method, as recommended by Applied Biosystems. Based on the Ct values, adipose expression of 18S rRNA was not affected by CORT treatment. The mtDNA analysis was performed as described [30], mtDNA was amplified using primers specific for the mitochondrial cytochrome b (Cyt B) gene and normalized to genomic DNA by amplification of the large ribosomal protein p 0 (36B4). All primers used in this study are shown in Table 1.

**Table 1:** Primer sequences used in this study.

Gene	Sequence (5′-3′)	Accession No.	Amplicon size (bp)
CytB	F: TGAGGGGGCTTCTCAGTAGA	NC_005089	118
	R: CTGTTTCGTGGAGGAAGAGG		
36B4	F: TGCCACACTCCATCATCAAT	NM_007475	240
	R: CGAAGAGACCGAATCCCATA		
PGC-1α	F: AATGCAGCGGTCTTAGCACT	NM_008904	193
	R: GTGTGAGGAGGGTCATCGTT		
NRF-1	F: CCACGTTGGATGAGTACACG	NM_001164226	120
	R: GCACCACATTCTCCAAAGGT		
CytC	F: CCAAATCTCCACGGTCTGTT	NM_007808	100
	R: TATCCTCTCCCCAGGTGATG		
IDH3α	F: GAGGTTTTGCTGGTGGTGTT	NM_029573	155
	R: TCCTCCTGGTCCTTGAATTG		
SIRT1	F: AGTTCCAGCCGTCTCTGTGT	NM_019812	198
	R: CTCCACGAACAGCTTCACAA		
PPARγ	F: CCCTGGCAAAGCATTTGTAT	NM_001127330	225
	R: GAAACTGGCACCCTTGAAAA		
CPT-1α	F: CCAGGCTACAGTGGGACATT	NM_013495	100
	R: AAGGAATGCAGGTCCACATC		
HSL	F: TGCTCTTCTTCGAGGGTGAT	NM_010719	183
	R: TCTCGTTGCGTTTGTAGTGC		
ATGL	F: TATCCGGTGGATGAAAGAGC	NM_001163689	112
	R: CAGTTCCACCTGCTCAGACA		
FAS	F: CCCTTGATGAAGAGGGATCA	NM_007988	115
	R: ACTCCACAGGTGGGAACAAG		
m18S rRNA	F: ACCGCGGTTCTATTTTGTTG	NR_003278	181
	R: TCGTCTTCGAAACTCCGACT		

### Mitochondrial biogenesis methods

Citrate synthase (EC Number: 2.3.3.1) activity in isolated mitochondria preparations of epididymal WAT was measured with spectrophotometer using citrate synthase kit (Sigma-Aldrich) following manufacturer's instructions. NAD^+^ and NADH levels in isolated mitochondria preparations were determined using the NAD/NADH assay kit purchased from BioAssay Systems (Hayward, CA, USA). Mitochondria were isolated using the Qproteome Mitochondria Isolation Kit (Qiagen).

### Statistical analysis

All statistical analyses were performed using SAS statistical packages (SAS Institute, Cary, NC, USA). Unpaired *t* test were applied to evaluate the comparisons between two groups. Data are presented as the mean ± SEM. Differences at *P*<0.05 are considered significant.

## Results

### Chronic CORT mildly decreases body weight

**Figure 1:**
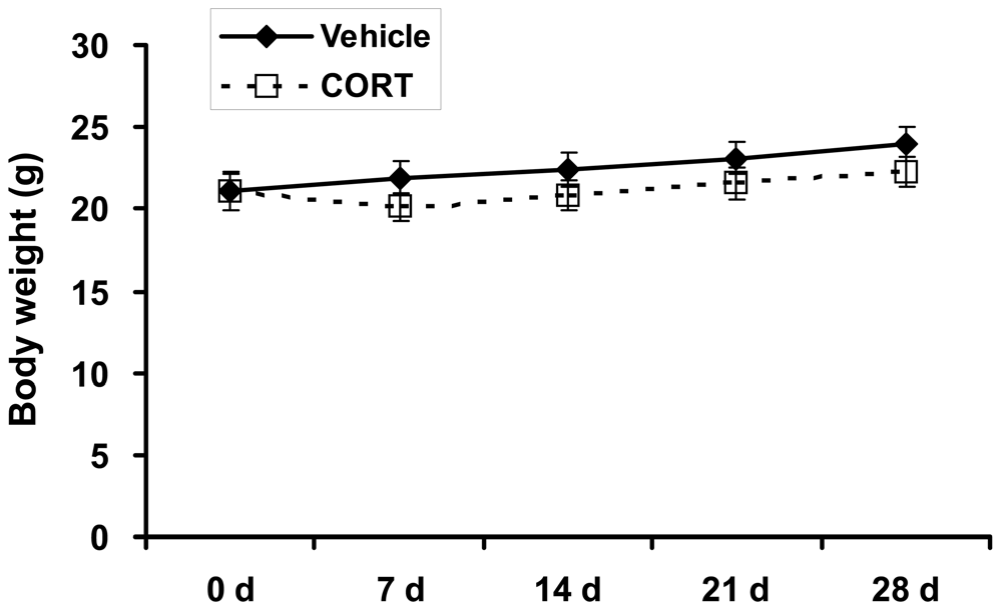
Effect of CORT on body weight of mice. Total body weight of mice treated with 100 µg/ml corticosterone (CORT) or vehicle during the experimental period. Data are expressed as group means ± SEM (n = 6 by group). * *P*<0.05 compared with vehicle.

We treated adult mice with 100 µg/ml of CORT through drinking water for 4 weeks to determine the effect of chronic CORT exposure on body weight change in mice. Mice in CORT group lost weight after the first week of treatment, but this did not reach statistical significance (*P*>0.05, Fig. 1). In the subsequent weeks, CORT-treated animals were smaller than vehicle-treated controls, but there is no significance (*P*>0.05, Fig. 1). CORT exposure slightly decreased the daily food intake (5.30±0.33 g vs 4.46±0.28 g, *P*>0.05). No significant change in daily drinking water intake was observed after CORT treatment (6.95±0.43 ml vs 7.13±0.15 ml, *P*>0.05).

### Chronic CORT exposure increases epididymal fat accumulation, but decreases adipocyte size

**Figure 2:**
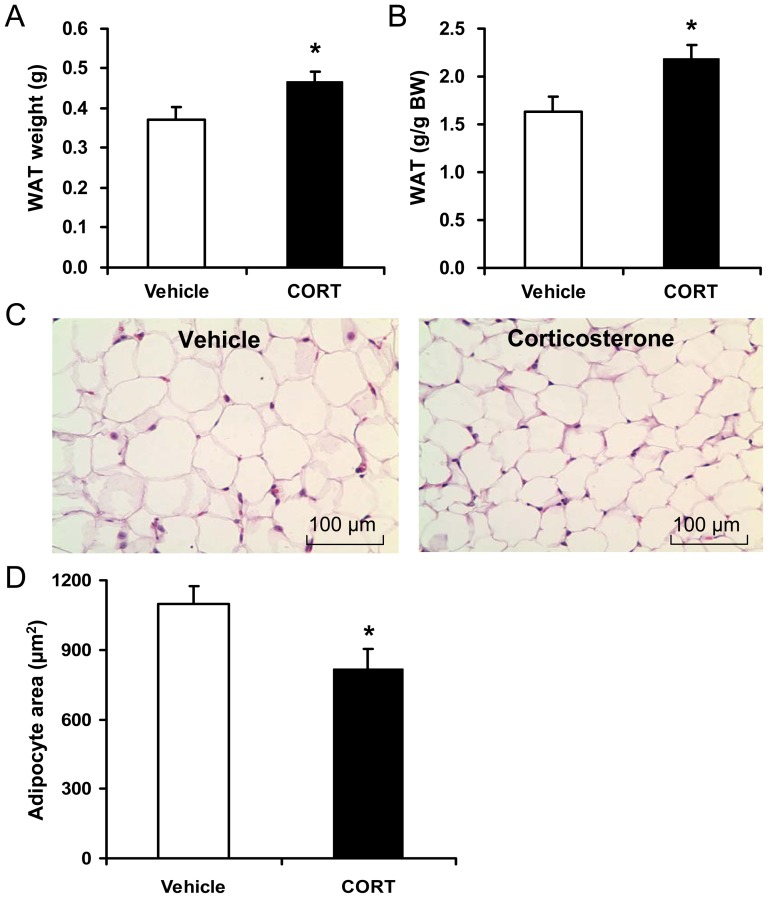
Effect of CORT on epididymal WAT weight and adipocyte size of mice. (**A**) Weight of epididymal white adipose tissue (WAT) and (**B**) relative contribution of epididymal WAT to total body weight in mice treated with 100 µg/ml corticosterone (CORT) or vehicle for 4 weeks. (**C**) Paraffin sections of epididymal WAT of mice treated with CORT or vehicle were stained with hematoxylin and eosin. Scale bar: 100 µm. (**D**) The average adipocyte cell area of epididymal fat in mice treated with CORT or vehicle. Data are expressed as group means ± SEM (n = 6 by group). * *P*<0.05 compared with vehicle.

The body composition of CORT-treated mice was subsequently analyzed to determine the relative amounts of WAT. CORT exposure significantly increased epididymal WAT mass (*P*<0.05, Fig. 2A), but did not alter retroperitoneal WAT (0.154±0.012 g vs 0.149±0.007 g, *P*>0.05) and inguinal WAT (0.287±0.048 g vs 0.267±0.043 g, *P*>0.05) weight compared with controls. Moreover, epididymal WAT made up a significantly larger proportion of their total body weight when compared to control mice (*P*<0.05, Fig. 2B). Interestingly, in the histological analysis, CORT-treated mice had significantly smaller adipocytes in their epididymal WAT compared with vehicle-treated animals (*P*<0.05, Fig. 2C and 2D), suggesting that the increased epididymal WAT mass is resulted from adipocyte hyperplasia.

### Chronic CORT exposure changes plasma hormone levels and lipid profile

To determine the circulating levels of hormones included in the regulation of lipid metabolism, plasma concentrations of CORT, insulin, leptin, and FGF21 were measured. Meanwhile, plasma triacylglycerols and NEFA levels were tested to determine the blood lipid profile. As expected, chronic CORT exposure induced significant increase in plasma CORT concentration compared with the control group (*P*<0.05, Table 2). CORT also significantly increased plasma concentrations of insulin, leptin and FGF21 when compared to control mice (*P*<0.05, Table 2). CORT group showed significantly higher triacylglycerols and NEFA levels compared with the vehicle group (*P*<0.05, Table 2).

**Table 2:** Effect of CORT on plasma hormone levels and lipid profile of mice.

	Vehicle	CORT
Corticosterone (ng/ml)	23.0±2.5	195.1±16.6*
Insulin (ng/ml)	1.52±0.21	7.49±0.15*
Leptin (ng/ml)	1.61±0.06	7.37±0.90*
FGF21 (pg/ml)	165.0±8.7	355.1±14.0*
Triacylglycerols (mM)	0.58±0.16	1.20±0.16*
NEFA (µM)	449.1±70.7	686.4±77.7*

Plasma concentrations of corticosterone, insulin, leptin, fibroblast growth factor 21 (FGF21), triacylglycerols and nonesterified fatty acids (NEFA) in mice treated with 100 µg/ml CORT or vehicle for 4 weeks.

Data are expressed as group means ± SEM (n = 6 by group).

* *P*<0.05 compared with vehicle.

### Chronic CORT exposure alters the expression of lipolysis and lipogenesis-related genes in epididymal WAT

**Figure 3:**
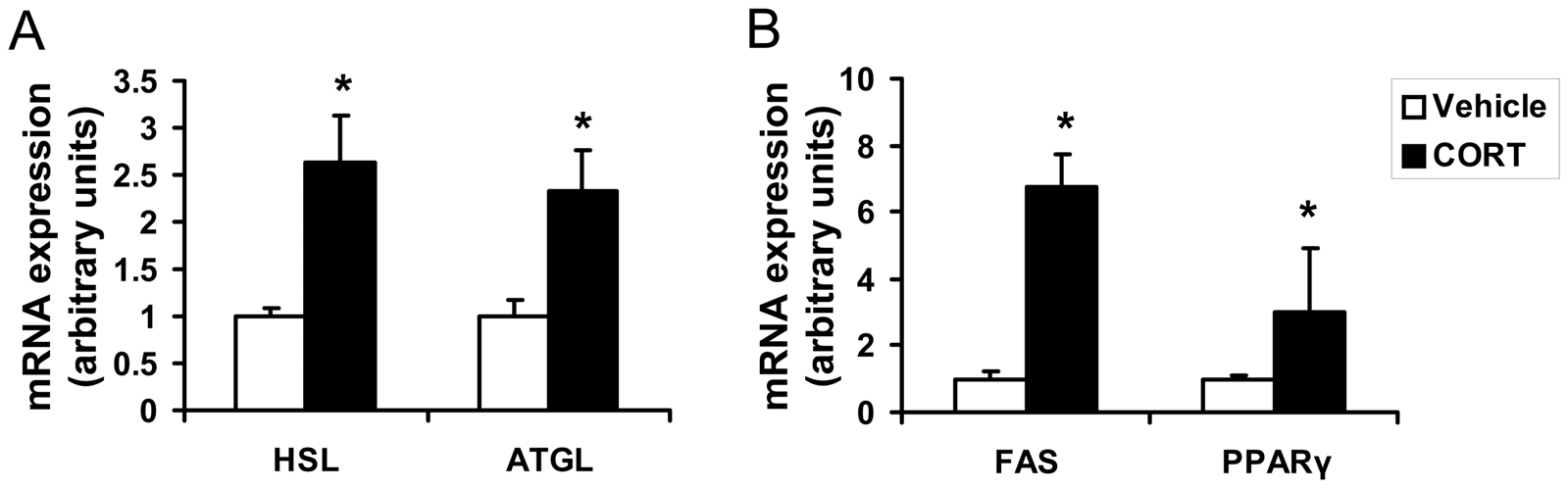
Effect of CORT on expressions of lipolytic and lipogenic genes in epididymal WAT of mice. (**A**) Relative expression of lipolysis-related genes, and (**B**) lipogenesis-related genes in epididymal white adipose tissue (WAT) of mice treated with 100 µg/ml CORT or vehicle for 4 weeks. Data are expressed as group means ± SEM (n = 6 by group). All the mRNA expression data are normalized to the house keeping gene (18S rRNA). * *P*<0.05 compared with vehicle.

We next determined the effects of chronic CORT exposure on the expression of genes related to lipolysis and lipogenesis. The expression of hormone-sensitive lipase *(HSL)* in epididymal WAT was significantly increased by CORT treatment (*P*<0.05, Fig. 3A). CORT also increased another lipolysis-related adipose triglyceride lipase *(ATGL)* expression in epididymal WAT compared with the control group (*P*<0.05, Fig. 3A). Interestingly, the mRNA expression of fatty acid synthase *(FAS)* and peroxisome proliferators-activated receptor γ *(PPARγ)*, the well known adipogenesis-related genes, were also increased 5.8 and 2.0 folds after 4-week CORT treatment, respectively (*P*<0.05, Fig. 3B).

### Chronic CORT exposure impairs mitochondrial biogenesis and functions in epididymal WAT

**Figure 4:**
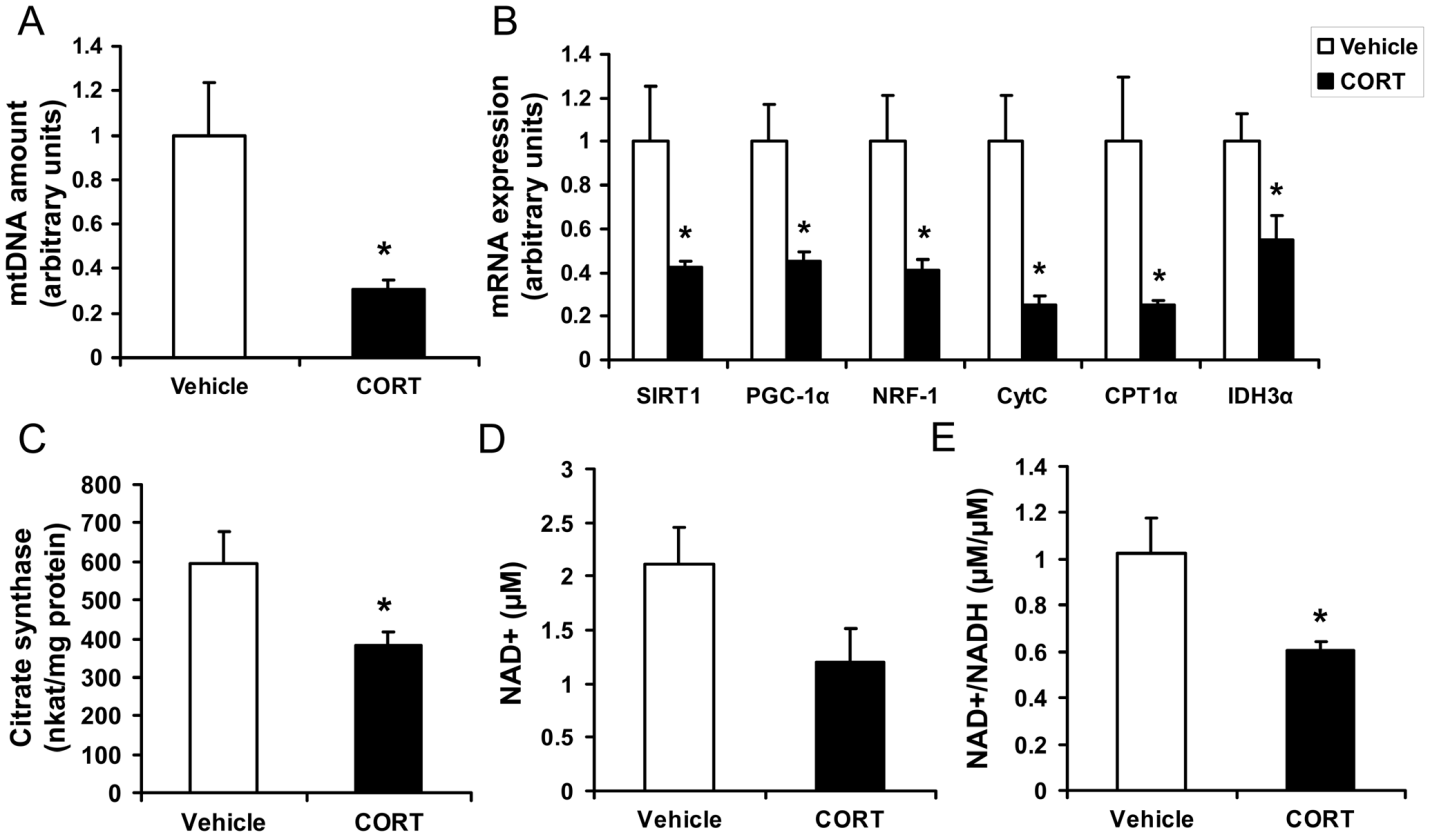
Effect of CORT on mitochondrial biogenesis and function in epididymal WAT of mice. (**A**) Relative abundance of mitochondrial DNA (mtDNA), (**B**) relative mRNA levels of mitochondrial genes responsible for mitochondrial biogenesis and function, (**C**) activity of citrate synthase (CS), (**D**) NAD^+^ levels, and (**E**) NAD^+^/NADH ratio in epididymal white adipose tissue (WAT) of mice treated with 100 µg/ml CORT or vehicle for 4 weeks. Data are expressed as group means ± SEM (n = 6 by group). All the mRNA expression data are normalized to the house keeping gene (18S rRNA), mtDNA is the relative amount to genomic DNA * *P*<0.05 compared with vehicle.

To determine the effects of chronic CORT exposure on mitochondrial biogenesis and functions, we measured mtDNA amount, mitochondrial gene expression, mitochondrial enzymatic activity, and NAD^+^ metabolism in epididymal WAT. The abundance of mtDNA in CORT-treated mice was significantly less than that in vehicle-treated controls (*P*<0.05, Fig. 4A). The mRNA levels of mitochondrial genes responsible for mitochondrial biogenesis and functions, including sirtuin 1 *(SIRT1)*, PPARγ coactivator 1α *(PGC1α)*, nuclear respiratory factor-1 *(NRF-1)*, cytochrome c *(CytC)*, carnitine palmitoyltransferase 1α *(CPT1α)* and isocitrate dehydrogenase 3α *(IDH3α)* were significantly decreased by CORT treatment when compared with control mice (*P*<0.05, Fig. 4B). Furthermore, CORT exposure induced a ∼40% decrease in citrate synthase activity (*P*<0.05, Fig. 4C). Though NADH level was not changed (data not shown), NAD^+^ level tended to be reduced (*P* = 0.09, Fig. 4D), and in turn intracellular NAD^+^/NADH ratio was remarkably decreased by CORT compared with vehicle (*P*<0.05, Fig. 4E).

### Chronic CORT exposure decreases ROS defense system in epididymal WAT

**Figure 5:**
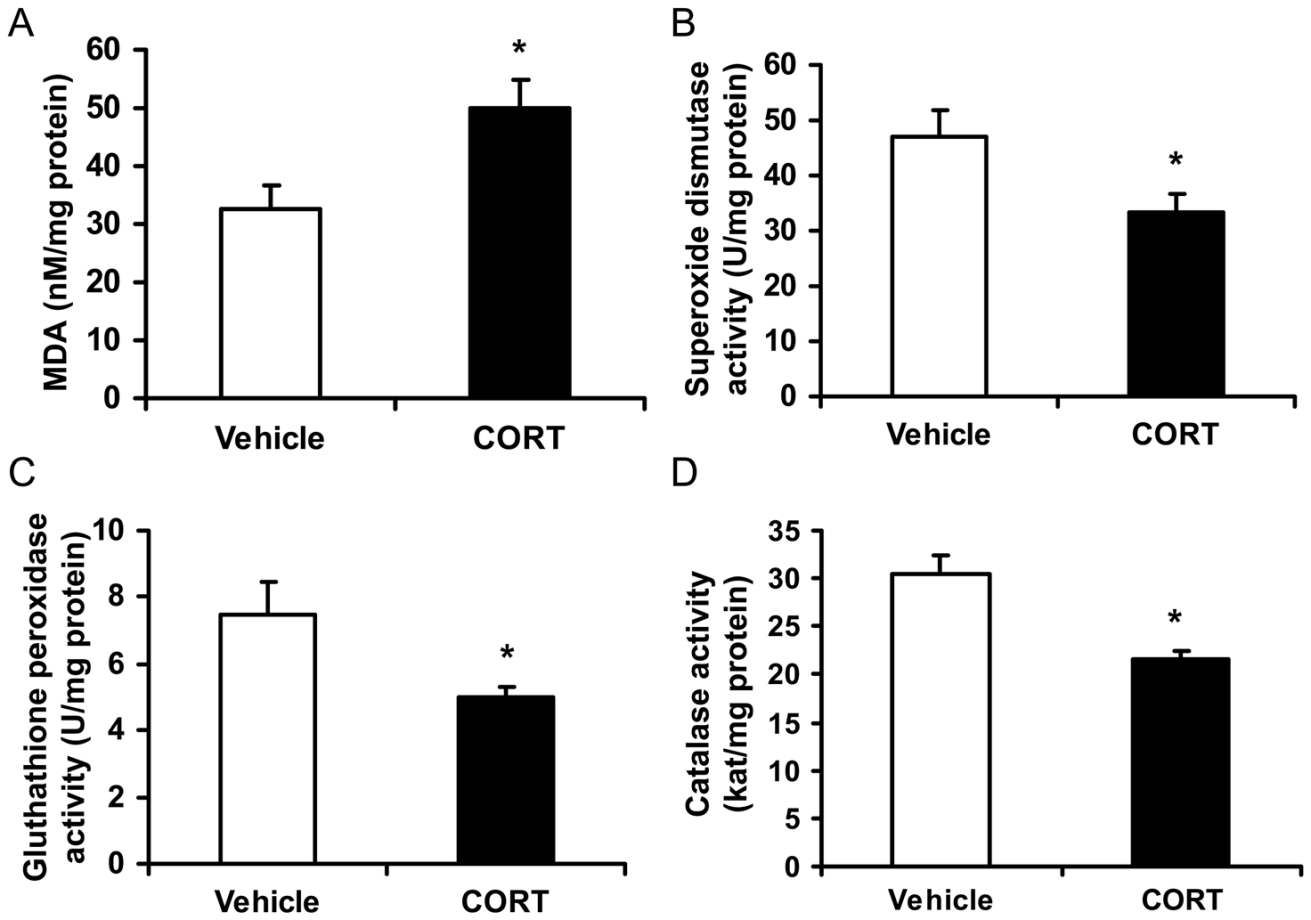
Effect of CORT on ROS defense system in epididymal WAT of mice. (**A**) Concentration of malondialdehyde (MDA), and activities of (**B**) superoxide dismutase, (**C**) gluthathione peroxidase, and (**D**) catalase in epididymal white adipose tissue (WAT) of mice treated with 100 µg/ml CORT or vehicle for 4 weeks. Data are expressed as group means ± SEM (n = 6 by group). * *P*<0.05 compared with vehicle.

We further measured the concentration of MDA and the activities of three major anti-oxidative enzymes (superoxide dismutase, gluthathione peroxidase and catalase) in epididymal WAT to determine the effects of chronic CORT exposure on ROS defense system. CORT group showed significantly higher MDA levels compared with controls (*P*<0.05, Fig. 5A), indicating more lipid peroxide accumulated in epididymal WAT of CORT-treated mice. Meanwhile, CORT treatment significantly decreased the activities of superoxide dismutase, gluthathione peroxidase and catalase in epididymal WAT of CORT-consumed mice when compared with vehicle-treated animals (*P*<0.05, Fig. 5B, 5C and 5D).

## Discussion

The metabolic syndrome presented in the patients suffering from either chronic endogenous hypercortisolism or exogenous GCs treatment contributes to the greater risk of cardiovascular diseases, diabetes and obesity, and reduces the life quality [5], [31]. In this study, our intriguing findings showed that chronic administration of CORT, the principal GC in mouse, through drinking water increases epididymal WAT amount without body weight gain, decreases epididymal adipocyte sizes, increases the expression of both lipogenic and lipolytic genes, impairs mitochondrial biogenesis, functions and antioxidant process when compared with control mice. Considering the crucial role of mitochondria in energy homeostasis, therefore, we have supposed that the epididymal adiposity appears during chronic GC exposure might be associated with GC-induced mitochondrial dysfunction.

Recent study has developed a potential model of the metabolic syndrome resulted from chronic CORT treatment [25]. This mouse model was applied in the present study to investigate the metabolic outcomes of mice under chronic CORT exposure. The CORT-treated mice averagely received 713 µg/d of CORT through drinking water according to the calculation from water intake. This oral dose is comparable with other studies conducted with subcutaneous GC pellets in male mice and female rats [32], [33]. As expected, plasma concentrations of CORT, insulin, leptin and FGF21 were elevated in mice treated with CORT. Although single plasma CORT levels give little information, previous studies using similar approach have demonstrated the hormone availability and endocrine physiology [25], [34]. Therefore, mouse exposed to CORT through drinking water could be a suitable model for human hypercortisolemia.

Surprisingly, we did not find rapid increases in body weight caused by chronic CORT treatment as previously published work [25]. However, as shown by numerous reports [8], [15], [25], [35]–[37], increased intra-abdominal rather than subcutaneous WAT together with elevated plasma triacylglycerols and NEFA levels in CORT-treated mice were also observed in this study, pointing to a sarcopenic phenotype. Active GC contributes to the sarcopenia, since excess GC induce hypoandrogenism and insulin resistance, both of which tend to decrease protein retention and induce loss of minerals from bone, muscle and other tissues [38], [39]. It's suggested that CORT-induced sacopenia leads to the relative increase in WAT at the expense of the rest of the body in the present study.

Another surprising finding in our study is that chronic CORT exposure reduced epididymal adipocyte size despite increased epididymal WAT weight. For exploring the mechanism underlying this phenomenon, the expression of genes related to lipolysis and lipogenesis were subsequently measured. In agreement with several recent studies [15], [40], we showed the up-regulation of these gene expression by chronic CORT exposure. A number of studies have also demonstrated either adipolytic or adipogenic effect of GC *in vivo* and *in vitrio*, depending on GC type, concentration, and duration [10], [41]–[44]. However, GC are most likely acting as a lipolytic or lipogenic factor through separate pathways. Lipolysis and lipogenesis are putatively concomitant in epididymal WAT under GC administration, and like our data, GC prefer to stimulate epididymal adipocyte hyperplasia rather than hypertrophy to induce central obesity.

Chronic CORT induces epididymal fat enlargement, and mitochondrial defects cause adiposity [45], but the relevant point is the linkage between these manifestations. Previous studies reported the present of glucocorticoid receptor (GR) and glucocorticoid response elements (GREs) in mitochondria [46], [47], suggesting glucocorticoids are able to directly act in mitochondria, and then regulate the energy metabolism, since mitochondria play key actors in global energy modulation [45]. Our results suggest that CORT impairs mitochondrial biogenesis and oxidative function in epididymal fat. In line with our data, a most recent report using rat pheochromocytoma PC12 cells has shown excessive glucocorticoid increases protein carbonylation, inhibits activities of mitochondrial complex I and superoxide dismutase [48]. Similarly, decreased activities of anti-oxidative enzymes and increased ROS levels were also observed in our CORT-treated epididymal WAT of mice, suggesting the failure of ROS defense system, which, on the other hand, is also an evidence of mitochondrial dysfunction [49]. Adipose tissue is peculiar in its defence systems, namely 11βHSD1, corticosteroid receptor regulation, and corticosteroid-binding globulin (CBG). Through the systems, GC action and availability could be modulated in adipose tissue [50], which make the mechanism by which CORT induces epididymal adiposity more complicated. However, it's clear that glucocorticoids elicit metabolic syndrome through alteration of ROS, damage to mitochondria, ER stress. Mitochondrial dysfunction also leads to abnormal lipid accumulation and the deterioration of the differentiation process [51]. These findings suggest that mitochondrial dysfunction contribute to CORT-induced epididymal adiposity, but the exact mechanism still warrants further investigation.

In conclusion, the present results indicate that chronic CORT administration induces epididymal adiposity and adipocyte hyperplasia. This is, at least in part, associate with mitochondrial dysfunction in epididymal WAT. Our work offers a potential explanation for the underlying mechanism of metabolic complications of excess glucocorticoid.
